# Paradoxical impact of memory on color appearance of faces

**DOI:** 10.1038/s41467-019-10073-8

**Published:** 2019-07-08

**Authors:** Maryam Hasantash, Rosa Lafer-Sousa, Arash Afraz, Bevil R. Conway

**Affiliations:** 1 Institute for Research in Fundamental Sciences, Tehran, P.O. Box 19395-5746 Iran; 2 Department of Brain and Cognitive Sciences, MIT, Cambridge, MA 02139 USA; 3 National Institute of Mental Health, NIH, Bethesda, MD 20892 USA; 4 National Eye Institute, NIH, Bethesda, MD 20892 USA

**Keywords:** Perception, Social behaviour, Colour vision, Object vision

## Abstract

What is color vision for? Here we compared the extent to which memory modulates color appearance of objects and faces. Participants matched the colors of stimuli illuminated by low-pressure sodium light, which renders scenes monochromatic. Matches for fruit were not predicted by stimulus identity. In contrast, matches for faces were predictable, but surprising: faces appeared green and looked sick. The paradoxical face-color percept could be explained by a Bayesian observer model constrained by efficient coding. The color-matching data suggest that the face-color prior is established by visual signals arising from the recently evolved L-M cone system, not the older S-cone channel. Taken together, the results show that when retinal mechanisms of color vision are impaired, the impact of memory on color perception is greatest for face color, supporting the idea that trichromatic color plays an important role in social communication.

## Abstract (web summary)

What is the function of color vision? Here, the authors show that when retinal mechanisms of color are impaired, memory has a paradoxical impact on color appearance that is selective for faces, providing evidence that color contributes to face encoding and social communication.

## Introduction

The benefits of color vision have been surprisingly difficult to pin down^1,2^. One popular idea is that color aids in foraging for food^3,4,5^. The main support for this hypothesis comes from an analysis of the spectral tuning of the cones and the chromatic signals of fruit^6,7^ and leaves^8^. But there are many surfaces besides fruit whose colors would be well discriminated by the cones, including artificial objects and faces^9^. Another idea is that color facilitates social communication about emotion, health, social status, and sex^10,11^ . Face color provides important cues to health, emotion, and attractiveness; and face context determines the meaning of the color of a face^12^. But the colors of other objects are similarly informative and determined by shape context. For example, a strawberry’s color determines its nutritive (and attractive) value, and its shape provides the context for this determination.

We sought to directly test the relative role of color in object and face perception by measuring the impact of shape on color appearance under viewing conditions that cause a loss-of-function of retinal mechanisms of color. Our approach was inspired by studies that probe memory colors using digital displays in which participants adjust images to appear achromatic^13^. In those studies, a banana that appears achromatic nonetheless retains some color as assessed with a colorimeter. One hypothesis is that the residual color is required to cancel a memory-induced color attributed to the banana shape. This logic implies that objectively achromatic renderings of color-diagnostic objects should appear somewhat tinged with their typical color, an idea that remains contentious^14^. Establishing the role of shape knowledge on color perception may depend on the vividness of the shape cues. For example, the impact of memory on color perception appears to be stronger when shape cues are enhanced^15,16^. To achieve the most vivid shape experience while impairing retinal mechanisms for color, we presented real-world stimuli in a room illuminated by monochromatic low-pressure sodium (LPS) light—such lighting causes a profound failure of color constancy and only variations in lightness can be perceived^17^ (Supplementary Fig. 1). Our goal was to measure under these conditions the colors people see in faces and objects, to evaluate the alternative hypotheses regarding the role of color in behavior. If memory modulates color perception, we predicted that objects with a diagnostic color, such as fruit and skin should have a subtle color corresponding to the normal colors of the stimuli (arbitrarily colored objects, such as Legos, serve as a control). The results were surprising. We found no clear evidence of the impact of memory on color appearance of the fruit, and a paradoxical impact of memory on the color appearance of faces: instead of appearing in their typical color, faces appeared green.

## Results

### Color matches under white and LPS light

**Fig. 1:**
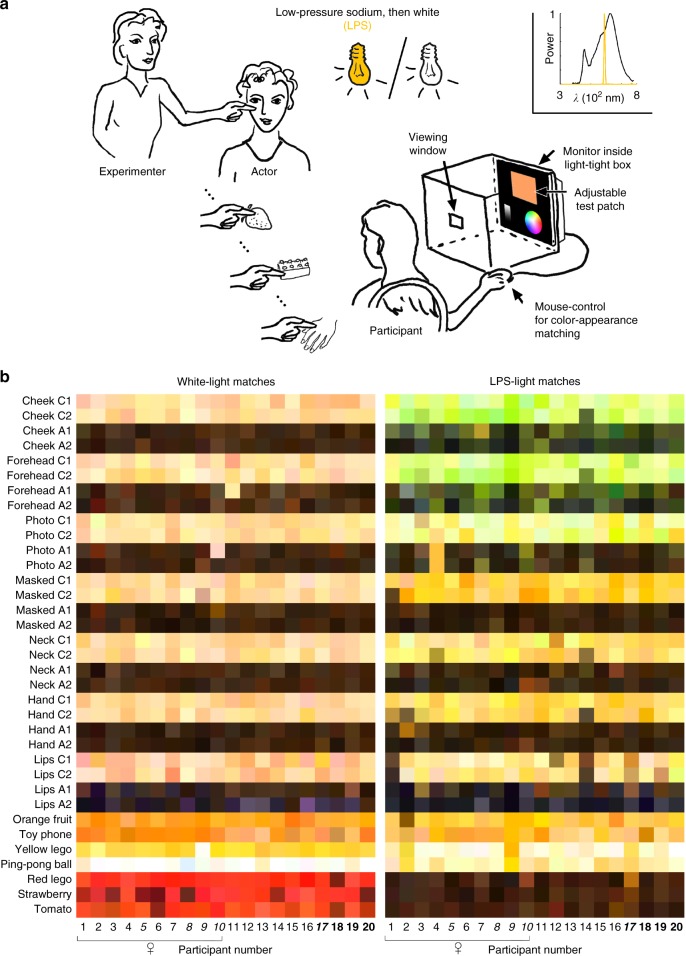
Color-matching real-world objects and skin under low-pressure sodium light, which impairs retinal mechanisms for color vision. **a** Participants (*N* = 20) used a computer to match the color of real-world objects (items listed in panel **b**) and skin (four female actors, 2 Caucasian, 2 African American), first illuminated by low-pressure sodium light and then broad-band white light (inset shows illuminant spectra). Each  participant was seated so that they could view the test stimuli shown to them by the experimenter, as well as a 2 × 2 cm viewing window in an otherwise light-tight box through which they could see a color-calibrated monitor (21.5-in. iMac computer, pixel resolution 1920 × 1080). Participants used a mouse to navigate a color-space disc and lightness strip, setting the hue, brightness, and chroma of the test patch to make the color match. Photographs and spectral measurements of objects are given in Supplementary Fig. 1. **b** Color-appearance matches made by all participants for the 35 test stimuli. The swatches are rendered using the RGB values as matched on a calibrated monitor (white point: *XYZ* = 87.4, 100, 57.7). Participant information: average age 27.5 (range 19–33); 10 female; 12 Caucasian; four Asian (italics); three African American (bold); one South-East Asian (italic bold). Source data are provided as a Source Data file

Twenty participants matched 35 stimuli, first under LPS light, and then under white light (Fig. 1a). The appearance of the matches (Fig. 1b) are not necessarily an accurate representation of the color appearance of the stimuli in the experimental conditions: first, the colors will depend on the calibration of the printer or display used to show the figure; second, the colors do not account for differences in the adaptation state under the different illumination conditions. Nonetheless, the figure shows that most participants matched the stimuli under white light as expected. For example, skin samples were pinkish or brownish (depending on the race of the actor); the strawberry and tomato were red; the orange fruit was orange; and the ping-pong ball was white. These color matches are comparable to colors that a separate group of participants gave when asked to match object colors only from memory (data not shown). Under LPS light most of the stimuli were matched as yellowish (varying in lightness, from yellow-orange to brown; Fig. 1b, right panel). Because the visual system would be adapted to the LPS light, it is likely that the stimuli did not appear as yellow to the participants as suggested by the yellowness of the matches reproduced in Fig. 1 (participants reported that most stimuli appeared depleted of color, consistent with prior reports^17^). Color matches for face stimuli were different from all other stimuli: faces were matched as green (Fig. 1b, right panel, top eight rows). Photographs of faces were matched with a slight green tinge, but not as green as the matches to real faces. After participants completed the matches under LPS light and before they proceeded to the tasks under white light, we asked participants to “tell us if you noticed anything about your color experience”. All participants stated that their color perception was not normal. Seventy percent of participants (*N* = 10/10 female, 4/10 male) reported that real faces looked green or otherwise sick (significantly more female than male participants, chi-square test, *p* = 0.003, Chi-square 8.57). More female subjects (*N* = 7/10) than male subjects (*N* = 2) reported that faces looked sick (chi-square test, *p* = 0.03, Chi-square 5.05). The paradoxical percept of face color under LPS light cannot be attributed to demand characteristics, since the color reports do not correspond to typical face color.

**Fig. 2:**
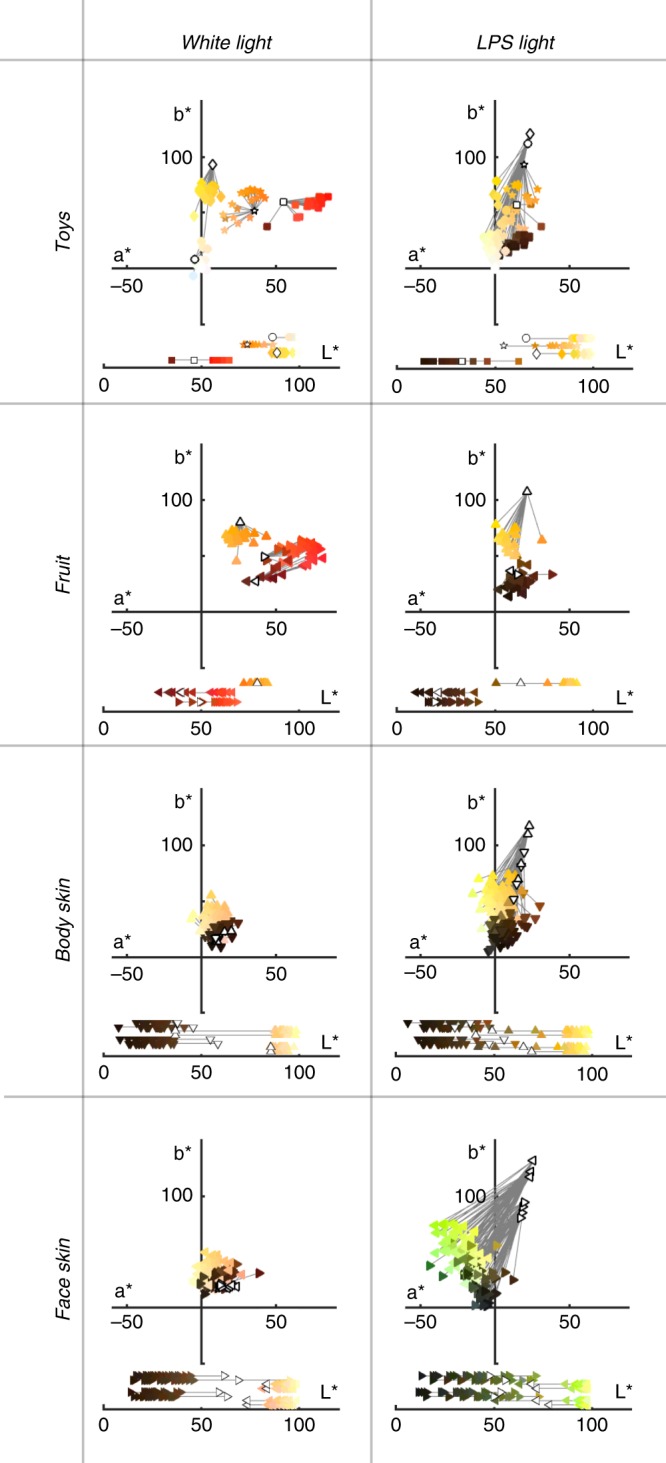
Color matches to real world objects and skin under white light and low-pressure sodium light. Participants’ (*N* = 20) color matches to toys (square = red Lego; diamond = yellow Lego; star = orange toy phone; circle = ping-pong ball), fruit (right-facing triangle = tomato; left-facing triangle = strawberry; upward triangle = orange), body skin (hand, neck of four actors; downward triangle = African American; upward triangle = Caucasian), and face skin (check, forehead of four actors; rightward triangle = African American; leftward triangle = Caucasian). Lines connect matched values (colored markers) to the spectroradiometric measurements (open markers). Spectroradiometric measurements were transformed to L*a*b* using the Macbeth white card under white light as the white point, CIE *XYZ* = [27.9446 25.0000 10.5134]). Matched values were transformed from RGB to CIE L*a*b* using the chromaticity coordinates of the monitor, gamma corrected. Source data are provided as a Source Data file

To quantify the color matches, the RGB values for each match were converted into L*a*b* color space (Supplementary Dataset 1). L*a*b* color space is designed to be perceptually uniform: the a*-axis shows the red-green perceptual dimension, which roughly aligns with the L-M cone-opponent axis; the b*-axis shows the blue-yellow dimension, which roughly aligns with the S-(L+M) cone-opponent axis. Plotted in these coordinates, the angle indicates hue (red, orange, yellow, etc.), while the vector length indicates saturation (chroma). As predicted, matches under white light to all objects (filled symbols, left panel Fig. 2) were close to the color values measured with a spectroradiometer (open symbols). Matches under the LPS light to the toys, fruit, and body skin were consistent with the spectrum of LPS light. Matches to face skin under LPS light showed a striking deviation towards negative a* values, corresponding to a greenish shift from the color of the LPS light (bottom right panel, Fig. 2). The lightness values of the color matches for Caucasian actors were shifted towards higher L* values compared to measured lightness values, while the lightness values of the color matches for African-American actors were shifted towards lower L* values; these race-dependent shifts in lightness matches were evident under both white light (two-tailed *t*-test: Caucasian, *p* = 10^−28^; African-American, *p* = 10^−16^) and LPS light (Caucasian, *p* = 10^−25^; African-American, *p* = 10^−3^) (Fig. 2; Supplementary Fig. 3). Matches under LPS light to all stimuli underestimated the chroma (saturation) values measured with a spectroradiometer, consistent with visual system adaptation to the spectrum of LPS.

**Fig. 3:**
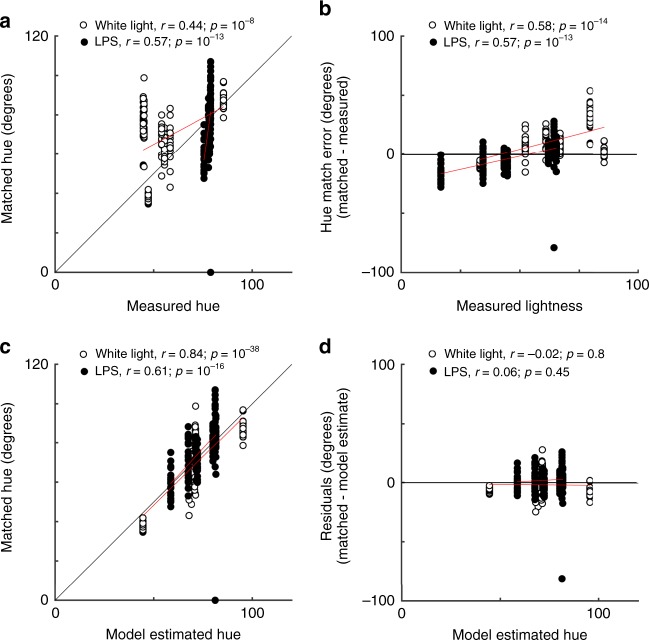
Estimating the impact of lightness and chroma on hue matching using matches to non-color-diagnostic objects. **a** Participants’ (*N* = 20) hue matches versus the measured hues for non-color-diagnostic stimuli (toys, masked forehead; CIE a*b* angle). Markers show results of matches made under white light (open symbols) and LPS light (solid symbols). **b** The difference between the angle of the matched hue and the measured hue versus measured lightness (white light, *r* = 0.58; LPS, *r* = 0.57). **c** Matched hue versus the estimated hue from the multivariate linear regression model. The model fit matched hue as predicted by measured hue, lightness, and chroma, for the non-color-diagnostic objects (white light, *r* = 0.84; LPS, *r* = 0.61). **d** Residuals of the model fit (matched—estimated) as a function of estimated hue (white light, *r* = −0.02; LPS, *r* = 0.06). Source data are provided as a Source Data file

Close inspection of Fig. 2 shows that the precise hue matches were correlated with measured lightness: Under both lighting conditions, objects of lower lightness were matched subtly redder compared to measured values, while objects with higher lightness were matched subtly yellower (darker data points tend to be to the right of their corresponding spectral measurements; lighter data points tend to be to the left). This interaction of lightness and hue perception has been described previously and reflects perceptual not cognitive mechanisms^18^. It has also been shown that chroma matches can be influenced by lightness^19^. To model the impact of lightness and chroma on matched hue we ran a multivariate linear regression. The model estimated matched values (of hue, lightness, and chroma) given measured values (of hue, lightness, and chroma), and was fit using data from objects whose color appearance is unlikely to be influenced by shape context (Legos, toy phone, masked forehead). To reconcile the mixture of circular and linear variables, we fit the model the using the a* and b* values as predictors, rather than the hue angle; the model’s predicted a* and b* values were then converted to hue angle. The discrepancy between the matched hues and the measured hues for these non-color-diagnostic objects (Fig. 3a) can be attributed in part to variation in the measured lightness of the stimuli (Fig. 3b). The slope of the correlation in Fig. 3b is very similar for matches under both LPS and white light; measured lightness accounted for little of the variance in measured hue under white light *r*^2^ = 0.12). The model provides an excellent fit—the model’s estimates are correlated with subjects’ matches (Fig. 3c) and the residual distributions are centered on zero and do not systematically vary with estimated hue (Fig. 3d). These results show that the model does a good job of accounting for the impact of measured lightness and chroma on hue matches.

**Fig. 4:**
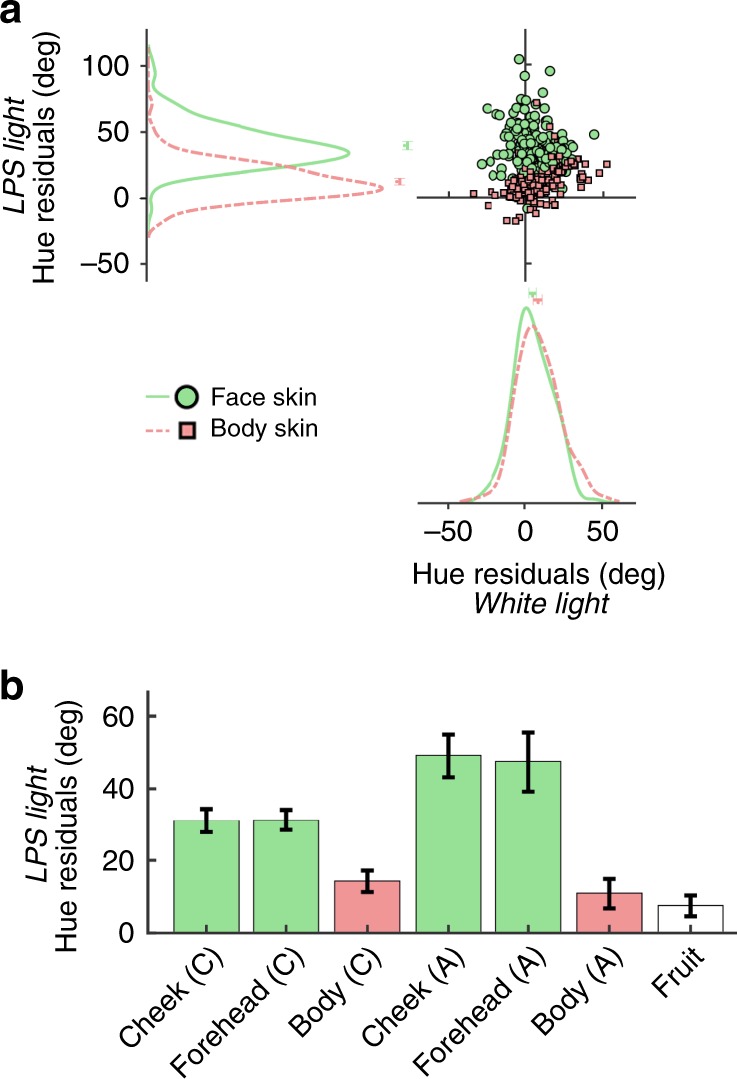
The impact of stimulus category on hue matches under low-pressure sodium light is greater for face skin than body skin or fruit. Hue matches to face skin (cheek, forehead), body skin (neck, hand), and fruit (tomato, strawberry, orange) were estimated from the multivariate linear regression model (see Fig. 3; (*N* = 20 participants). **a** Hue angle not explained by the model (residuals) for skin matches under LPS light versus white light (160 face-skin matches, 160 body-skin matches: 2 face-skin regions; 2 body-skin regions; 4 actors; 20 participants). Markers show averages and 99% C.I. **b** Residuals for hue matches under LPS light for fruit, and for face skin (cheek, forehead) and body skin (neck and hand, combined) of the two races tested (Caucasian, C; African American, A). Error bars show 99% C.I. Source data are provided as a Source Data file

Residuals obtained by applying the model (trained on non-color-diagnostic objects) to the data obtained for color-diagnostic stimuli (fruit, face skin, body skin) would provide evidence of the impact of object-color knowledge on color appearance. Residuals for the matches made under white light for body and face skin are centered near zero (average [99% CI] for face skin: 5.3 [2.7, 7.8]; body skin: 8.8 [5.9, 11.2]), Fig. 4a). Residuals of the matches made under LPS light for body skin are also centered near zero (12.6 [10.1 15.4], but residuals of the matches under LPS light for face skin are shifted away from zero (39.8 [36.6, 43.0]). The difference between the residuals for matches to face and body under LPS light are different from the corresponding measurements under white light (paired *t*-test, *p* = 10^−37^; Fig. 4a). Unlike the residuals of the matches to face skin and body skin under white light, the residuals for matches to lips under white light had high variance (average [99% CI] for Caucasian lips: 38.15 [32.2, 43.5]; African American lips: −24.6 [−52.3, −0.6] (*F*-test, *p* = 10^−78^). Residuals for matches under LPS light are higher for face stimuli, regardless of race, than for fruit (Fig. 4b). Residuals for matches to fruit and body skin had a relatively small magnitude but were nonetheless different from zero, which is compatible with the hypothesis that color matches to these stimuli were modulated by memory^13,15,16^. But we cannot rule out alternative explanations, namely that the small-magnitude residuals for fruit and body skin indicate that the model is imperfect and/or color matches to these stimuli reflect a subtle demand characteristic. The analysis of the residuals showed subtle differences in the matches to Caucasian versus African-American skin (Fig. 4b), which probably reflects the systematic impact of race on lightness matches (Fig. 2, Supplementary Fig. 3). A three-way ANOVA of lighting condition (white light versus LPS light), skin type (face skin versus body skin), and race (African-American versus Caucasian) uncovered a main effect of lighting (*p* = 10^−62^); a main effect of skin type (*p* = 10^−28^); no main effect of race (*p* = 0.5); an interaction of lighting and skin type (*p* = 10^−43^); and a three-way interaction of lighting, skin type, and race (*p* = 10^−9^). Despite the sex differences in the reports made by participants, there was no sex difference in the residuals for the color matches to faces (two-tailed *t*-test: LPS, *p* = 0.16; white light, *p* = 0.95)—this shows that male and female participants were equally likely to see faces as green under LPS light but were not equally likely to tell us about it.

**Fig. 5:**
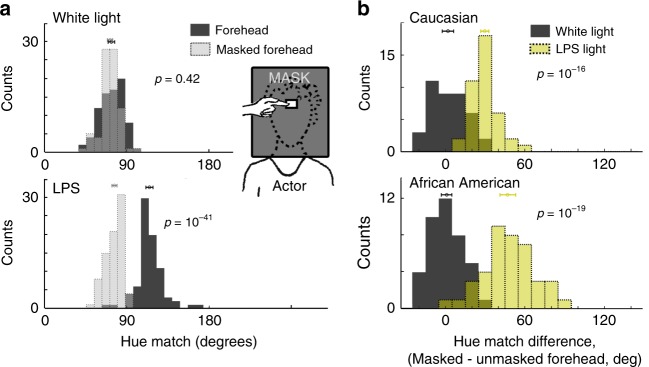
Paradoxical color matches for face skin depends on face-shape information and is not race-dependent. **a** Histograms showing the angle of the color matches for forehead and masked forehead under white light (top) and low-pressure sodium light (bottom) (*N* = 20 participants; matches made to four actors: two Caucasian, two African American). Color matches in the masked condition were made by holding a sheet of black cardboard in front of the actor’s face, with a 1.5 cm × 2 cm aperture over the forehead. Masking the face context did not change the hue match under white light (*p* = 0.42) but had a large impact on the color match under LPS light (*p* = 10^−41^; *p-*values obtained by *t*-test not assuming equal variance; markers show average values, and error bars are 99% C.I.). **b** Histograms showing the difference in hue angle for color matches to forehead versus masked forehead, under white light versus LPS light, for Caucasian skin (top) and African American skin (bottom). The two distributions for each race do not have the same mean (Caucasian *p* = 10^−16^; African American *p* = 10^−19^; markers and error bars as for panel **a**). Source data are provided as a Source Data file

**Fig. 6:**
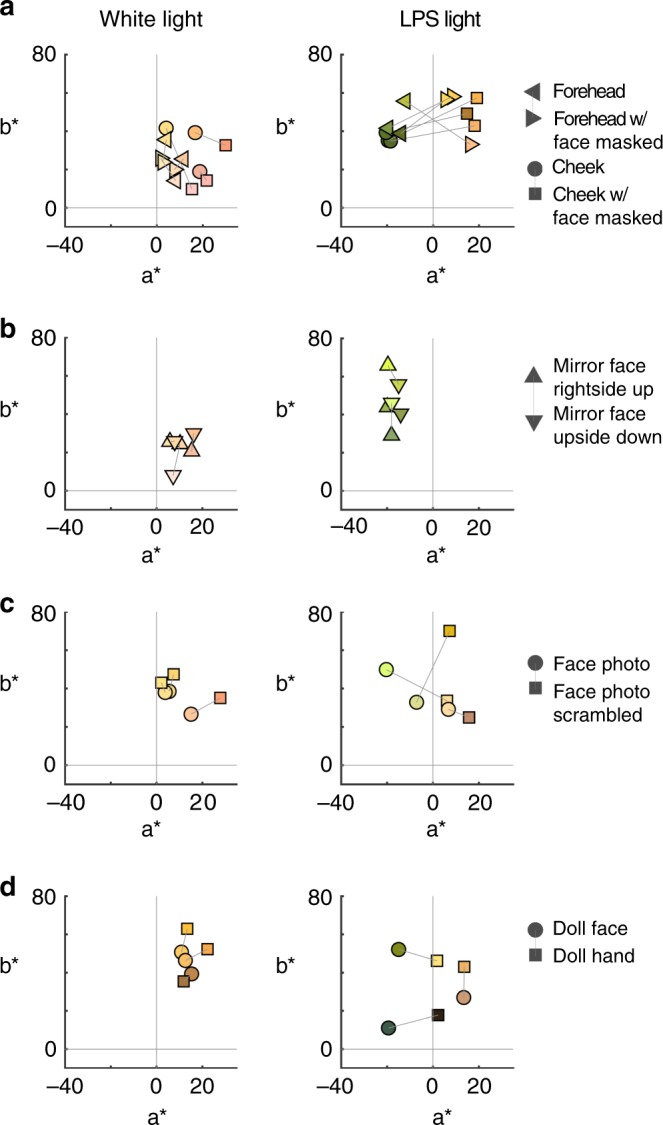
Paradoxical color matches for faces are evident in mirror inverted faces, photos, and a doll face. Color matches under white light and low-pressure sodium light; corresponding stimuli are connected by a line (see key). Data were collected in three participants (#5, 7, 19, in Fig. 1). **a** Forehead and masked-forehead, and cheek and masked cheek. Matches to the masked condition are to the right of the matches to the unmasked condition. **b** Faces seen upright and upside-down in a mirror. For the upside-down condition, matches were made by looking down at a mirror view of the face. **c** Photograph of a Caucasian female face and scrambled photograph of the face. **d** Doll face and doll hand; the doll hand did not show a paradoxical color match for any participant. Symbol color corresponds to the color of the match. Source data are provided as a Source Data file

The analysis of the residuals shown in Fig. 4a suggests that the paradoxical color appearance of faces is determined by face context not stimulus material. But body skin can be slightly different from face skin; for example, body skin might have different texture or vascularization. To rule out these possible material differences as an explanation for the paradoxical color matches of faces, we compared the hue matches made to the identical stimulus—the forehead region—with and without face context (Fig. 5). Matches were made to a patch of forehead with the rest of the face masked and to the same region without the mask. We found no impact of face context on hue matches under white light (Fig. 5a, top), but a large effect of face context on hue matches under LPS light (Fig. 5a, bottom). This result was clear for both races tested (Fig. 5b) and shows that the paradoxical color appearance is caused by face context. Because the paradoxical color matches for faces were only partially evident in photographs of faces (Fig. 1), we quantified with an ANOVA the impact of face (cheek, unmasked forehead, photo) versus non-face (hand, neck, masked forehead) and three dimensionality (cheek, unmasked forehead, neck, hand) versus two dimensionality (photo and masked forehead) on the hue matches under LPS light. The results showed a main effect of face (*p* = 10^−68^), a main effect of 3-D cues (*p* = 10^−23^), and an interaction of the two factors (*p* = 10^−11^). These results show that the paradoxical color matches for faces are dependent on face-shape context enhanced by having richer shape cues. In three participants we measured color matches to real faces mirror reversed and upside down, and to a doll’s face and hand. The main results were replicated in these participants (Fig. 6a). The paradoxical color matches for faces were evident in real faces mirror reversed and upside down (Fig. 6b); weakly in photographs but not in scrambled photographs (Fig. 6c); and in a doll’s face but not in a doll’s hand (Fig. 6d). These results confirm that the green-face effect was not dependent on a specific reflective or texture property of skin and was enhanced by having more vivid shape cues.

**Fig. 7:**
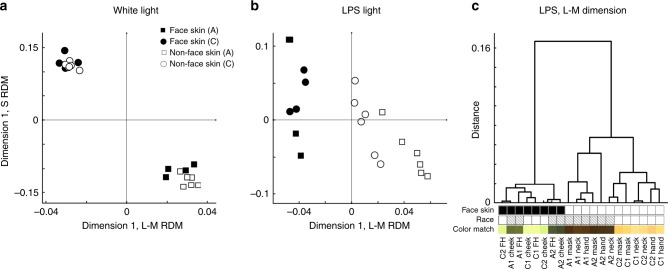
Paradoxical color matches for faces is encoded by the more recently evolved (L–M) system. **a** Color matches for skin were evaluated for the extent to which they modulated the L–M versus S cone systems; these values were then subjected to a hierarchical clustering algorithm to determine the relative similarity among matches across the various skin samples; data from 20 participants. The plots show multidimensional scaling (MDS) relating the first dimension of the representational dissimilarity matrix (RDM) for the S component against the first dimension of the RDM for the L–M component, for matches under white light. Matches were made to face skin: cheek and forehead; and non-face skin: masked forehead, neck, hand. Face skin and non-face skin are not separable with either the L–M or the S dimensions. **b** As for panel **a**, but for matches made under low-pressure sodium light. Face skin and non-face skin are separable with the L–M dimension but not the S dimension; the L–M dimension also separates body skin by race. **c** Dendrogram showing the relative dissimilarity distances of the L–M component of the matches under LPS light. The main branch separates face skin from non-face skin; a secondary branch separates race among matches to body skin, but not face skin. Source data are provided as a Source Data file

The color matches made under LPS light provide information about the signals that the brain uses to form knowledge about skin color. Color matches to face skin versus non-face skin were indistinguishable under white light (Fig. 7a), but they were distinguishable under LPS light by the extent to which they modulated the L–M color axis (Fig. 7b, c). This suggests that the memory color of faces is encoded by signals that modulate a differential L-cone versus M-cone signal. The L–M system, which defines trichromacy and arose relatively recently in primate evolution^5^, relays information about health, sex, emotion, and attractiveness^20,21^—such information is dynamic and independent of face identity^22^. The paradoxical color matches under LPS light imply that perception of face color is weighted towards dynamic features—the emotion or health of a face—rather than stable properties such as identity. Taken together, the results are consistent with the idea that selective pressures related to social cognition fueled the evolution of trichromacy in our primate ancestors.

## Discussion

The experiments described here probe the impact of memory on color perception and uncover a special role of color in face perception. Consistent with the observation that scenes under LPS light impair retinal mechanisms for color^17^, color matches under LPS light for arbitrarily colored objects were not predicted by colors seen under white light. Color matches for fruit under LPS light were also not predicted by stimulus identity: for example, knowledge that a strawberry is red did not cause participants to match the LPS-illuminated strawberry as red. These results show that cognition does not always hold sway over color appearance. But unexpectedly, color matches under LPS light to one class of stimuli, face skin, were predictable, although surprising: all participants matched faces green. Furthermore, most participants (female>male) reported that faces looked green or appeared sick, showing that the modulation by memory of face color does not remain unconscious. This paradoxical percept was evident for faces of both races tested, was abolished when the face context was masked, and was not observed for matches made to body skin. The results lead to three conclusions. First, the brain has a strong prior specifically for the color of skin, which triggers a prediction-error signal, possibly diagnostic of sickness, when violated in the context of rich face-shape information. Second, trichromatic color plays an especially important role in social communication. Third, cognition can influence perception, refuting notions to the contrary^14^. Memory not only modulated perception of face color, but also impacted the lightness matches made to skin reflecting knowledge about race, confirming prior results^23^ (Supplementary Fig. 3).

**Fig. 8:**
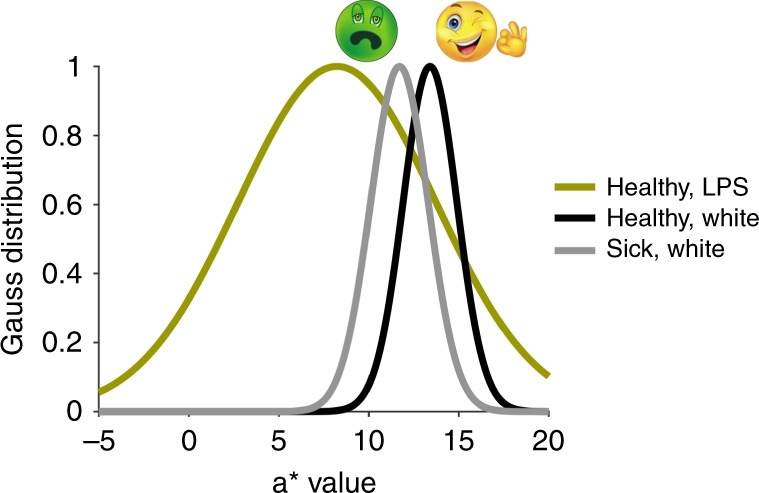
Probability of a* values given health or sickness and lighting condition. The probability distribution for healthy skin under sodium light was generated using participant matches of masked forehead. Gaussian distributions for healthy and sick faces were obtained from the mean and standard deviation of measurements of cheek and forehead provided by Henderson et al.^25^. Source data are provided as a Source Data file. Smiley emoticon with OK sign created by Tigatelu with Dreamstime.com. All rights reserved. Sick Emoji created by B.R.C.

Why do faces under LPS light look green? LPS light leaves intact rich shape cues, making it inescapable that the face is real. In the context of a real face, the peculiar spectral signals cannot be discounted with a trivial explanation, unlike the color in a photograph or digital reproduction, which can be attributed to the way the image was generated. Under LPS light, the spectral signals from skin are characterized by a decrement in redness (Fig. 8); a similar decrement accompanies many illnesses^24,25^, caused by sympathetic vasoconstriction of superficial blood vessels or anemia. We suspect that participants attribute the peculiar chromatic signal to sickness as the most likely explanation, which would explain why many participants described LPS-illuminated faces as sick-looking. The chromatic signals of LPS-illuminated skin violate a prior about healthy skin, breaching a naturalness constraint^26^. But why should this breach cause a green appearance?

The decrement in redness for either LPS-illuminated faces or sick faces does not yield negative a* values—colorimetrically the signal is still reddish. The green appearance of faces under LPS light is paradoxical: it is an exaggeration of the greenward shift of the received signal compared to the prior. A standard Bayesian account argues that perception is biased towards the prior, not away from it. Yet circumstances in which percepts are biased away from a prior have been described in other domains, for example orientation, biological motion, and size/weight^27–29^. Although these phenomena were originally thought to reflect anti-Bayesian processes, they can be accounted for by a Bayesian observer model constrained by efficient coding, in which the statistics of the natural environment shape both the encoding and decoding of sensory information^30^. In this framework, there is a nonlinear mapping between stimulus and sensory representation that results in repulsive perceptual estimates for stimuli that sufficiently violate sensory expectations. One can think of this repulsion as a form of contrast-enhancement that promotes behaviorally important categorization^31^. According to this interpretation, the exaggeration of prediction errors provides evidence of a computational objective of the visual system: here, the vital distinction between health and sickness. The present results suggest the visual system accomplishes this objective by encoding the statistics of healthy skin. The relative importance of skin color to human behavior is well known: humans have precise color preferences for skin, do not tolerate poor color reproduction of skin^32–35^, are especially sensitive to changes in facial redness^21^, and can use skin color towards color constancy^36,37^. The results here predict that any circumstance in which the chromatic signals arising from the face deviate from the face-color prior (and cannot be explained away by any more likely explanation) should appear peculiar. Consistent with this prediction, sick faces often appear green^38^, evident in emojis (Fig. 8). Moreover, repulsive biases should scale with prediction error. Faces under LPS light incur larger red decrements than do most sick faces, which may explain why faces under LPS light are matched green by almost all observers.

It is widely thought that color and face perception are handled by separate neural circuits^39^. The fact that the paradoxical color percepts reported here depend on face shape implies that color and face shape are processed by some common neural substrate somewhere in the brain. The systematic relationship of face-biased and color-biased regions in the cerebral cortex, and their convergence at the anterior pole of inferior-temporal cortex^22,40^, may provide clues to this neural substrate. In addition, the results and interpretation described here predict the existence of neural operations dedicated to encoding skin color, and in particular, neural tuning/populations biased toward skin color priors. This prediction is supported by evidence of a broad bias in the ventral visual pathway towards warm (L>M) colors^9^, and preliminary work showing an L>M color-bias specifically in face-selective neurons^41^.

Regardless of the mechanism, the results show for the first time that the brain assigns special weight to the color signals from faces compared to color signals from other objects including ripe fruit. This is an important finding because it has been difficult to disentangle competing accounts of the evolutionary pressures that selected for and then maintained trichromatic color vision^4,42^. Among mammals, trichromacy is found routinely only in old world primates, such as macaque monkeys and humans^5^; trichromacy is also found in new world primates, but only among females, with a notable exception^43^. The differences in color vision genetics between new world primates and old world primates suggests that routine trichromacy evolved after the time that these species diverged, ~30-40 million years ago^5^. It is unsettled whether the allelic variation that gave rise to trichromacy in new world and old world primates arose before the divergence of these species^5^ or after^44^. Nonetheless, among old world primates, trichromatic vision is presumably under strong selective pressure since rates of red-green colorblindness are vanishingly low in wild populations^45^. Was the selective advantage for trichromacy related to foraging or social communication, or some other behavior? The spectral tuning of L and M cones allows the discrimination of ripe fruit^6–7^, nutritious leaves^8^, and objects from backgrounds more generally^9^. But despite long-standing dogma^3,4,5^, there appears to be little advantage conferred by trichromacy on foraging among wild primate populations^46,47,48^, which promotes an alternative account of the selective advantage of trichromacy, such as encoding chromatic signals associated with health, sex, social status, and emotion^20,49,50,51^, especially in the face^12,52^. This hypothesis is supported by an analysis of color vision, skin color, pelage color, and social systems traits among primates, which suggests that the potential for trichromatic vision existed before the evolution of traits used in social communication, but once trichromacy evolved, it promoted the evolution of red traits  (including hair loss on the face) through sexual selection^53^. The results presented here do not refute a role of color in detecting, discriminating, recognizing, and remembering objects, scenes, or fruit^2,11,54^, but they help resolve the relative value of color by showing that trichromatic color signals from the face are especially important for behavior. The results lend weight to the idea that regardless of the selective pressures that drove the initial evolution of trichromacy in old world primates, trichromacy has been maintained because of its role in social signaling. 

## Methods

### Color matching procedure

Twenty people with normal color vision (tested with Ishihara plates) participated in the experiment. Each participant matched the color appearance of seven real-world objects including four artificially colored toys (orange phone, red Lego, yellow Lego, white ping-pong ball), three ripe fruits (strawberry, tomato, orange), and various skin regions of four actors (all female; two Caucasian, two African American; no makeup) (Fig. 1a). Test objects were chosen to have the same palette as skin. The Legos and toy phone were included because they should not give rise to shape-dependent color percepts since their colors are arbitrary. All experimental procedures were approved by the Wellesley College Institutional Review Boards, the Massachusetts Institute of Technology Committee on the Use of Humans as Experimental Subjects, and the National Institutes of Health Intramural Institute Clinical Research Review Committee. All participants and actors provided written informed consent and were compensated financially for their involvement. The two actors whose photographs are shown in Supplementary Fig. 1 provided written consent to publish their photographs.

Color appearance matches were made using a calibrated computer monitor encased in a light-tight box, inside the testing room. Participants could see the monitor by lifting a small black cloth to reveal a 2 cm^2^ viewing window. The monitor showed a color-space disc and a lightness bar that the participant could use to adjust a central patch to make their selection with a computer mouse. The monitor was otherwise black, and the color of the test patch was random at the onset of testing. Participants were instructed to match the color of the patch as accurately as possible to the color appearance of the test stimuli, and not to match the color of the stimuli as recalled from memory. We were mindful of the potential for demand characteristics to influence the results: participants might set the test patch to match the typical colors of the stimuli even if they perceived the stimuli to be achromatic. Our initial aim was to quantitatively compare matches for fruit, face skin, and body skin, with the assumption that demand characteristics would equally impact judgments of all stimuli.  The results provide evidence against a role of demand characteristics under our experimental conditions.

The participants did the matching twice: first while the testing room was illuminated with LPS light, and then again after the room light was switched to normal white light (participants adapted to the illumination for ~7 min). Participants were tested first under LPS light to prevent short-term recall of color matches made under white light. Test stimuli were presented in a unique order for each participant (Supplementary Dataset 2) at ~1 m viewing distance. The precise region to be matched was indicated with a lightly drawn circle (for the objects) or by pointing to regions on the actors (forehead, lips, neck, and back of the hand). Participants also color-matched the forehead of each actor in a photograph and the actors’ foreheads while masking the rest of the face with black paper (~1 cm diameter aperture). All participants matched the same region of each stimulus, and the lighting on the stimuli was consistent across participants: stimuli were placed at a set location on a table, at a fixed distance from the light sources; actors were seated with their faces at a fixed orientation and gaze angle with respect to the participant. The actors entered the testing room one at a time. The participant’s adaptation state was preserved during transitions by having the participant close their eyes and by keeping the anteroom dark. Participants performed the color-matching tasks reliably and consistently; Supplementary Fig. 3 shows test–retest reliability measures for three subjects, tested several months apart. An initial pilot experiment involving separate participants and actors yielded similar conclusions and provided the basis for the experimental approach reported here.

### Data analysis

The visual system implements color constancy operations that correct for the spectral bias in the illuminant. Color constancy can be almost perfect^55^, but fails when the illuminant is monochromatic, as under LPS light^17^. The consequence is that color cards viewed under LPS light have an eerie quality: they are tinged with the color of the light, but as Boynton et al. describe^17^ only variations in lightness are perceived. The perception of brightness, depth, perspective, shape, shading, and motion remain intact. The luminance distribution across objects under LPS light was comparable to that under white light, and there was no systematic difference in the luminance distribution under these two illuminants for faces compared to other objects we tested (Supplementary Fig. 1). Adaptation algorithms that predict color appearance given the illuminant are imperfect and get worse the further an illuminant is from neutral^56^. Because of these constraints, color-correction algorithms are not able to estimate color appearance under the LPS light. Spectral measurements of objects under both white light and LPS light were transformed from CIE *XYZ* to CIE L*a*b* using spectral measurements of the Macbeth white card under white light as the white point (Supplementary Fig. 1). In this report, we present two analyses of subjects’ color matches. First, we analyzed the raw color matches obtained on the calibrated matching monitor (see Fig. 1), transformed from RGB to CIE L*a*b* using the measured chromaticity coordinates and luminance curves of the monitor’s R, G, B channels (see Figs. 2, 5, 6, and 7). The chromaticity values and luminance curves of the matching monitor were obtained with a spectroradiometer (PR655, Chatsworth, CA) and the monitor was gamma-corrected. Second, we estimated errors in hue matches attributed to lightness and chroma, and analyzed the residuals (see Figs. 3 and 4). These residuals provide an estimate of the impact of color-shape knowledge (priors) on color perception. We also did an analysis in which we empirically estimated the adaptation state for each participant under each experimental condition by having participants match a white ping-pong ball. This approach allowed us to control for variability among our participants in how each person’s visual system adjusted to the LPS light; the main results are the same using this approach. But because the white points are slightly different for each participant, and the impact on the gamut is not trivial to compute, we only present the results and analysis using the color space in which the matches were obtained. Throughout the figures, confidence intervals were generated by 1000 bootstraps.

### Reporting summary

Further information on research design is available in the Nature Research Reporting Summary linked to this article.

## Supplementary information

Supplementary Information

Reporting Summary

Description of Additional Supplementary Information

Supplementary Dataset 1

Supplementary Dataset 2

Source Data

## Data Availability

All data and analysis code are available on a public-accessible website (https://neicommons.nei.nih.gov/#/facecolor).
