# Supplementary material for: Paradoxical impact of memory on color appearance of faces
Source: Nat Commun. 2019 Jul 8;10:3010. doi: 10.1038/s41467-019-10073-8 (PMC6614425; doi:10.1038/s41467-019-10073-8)
Supplement: Supplementary file 1 — Supplementary Information [file 41467_2019_10073_MOESM1_ESM.pdf]

Supplementary Information

Contents:

Supplementary Figure 1
Supplementary Figure 2
Supplementary Figure 3
Supplementary Figure 4

SOURCE_DATA.xls

SOURCE_DATA.xls

Source data are provided as a Source Data file. This file contains the raw and processed data for all analyses presented. For each Figure in the publication, the source data file contains a corresponding data tab/sheet.

Complete analysis code is available at: **https://neicommons.nei.nih.gov/#/facecolor**

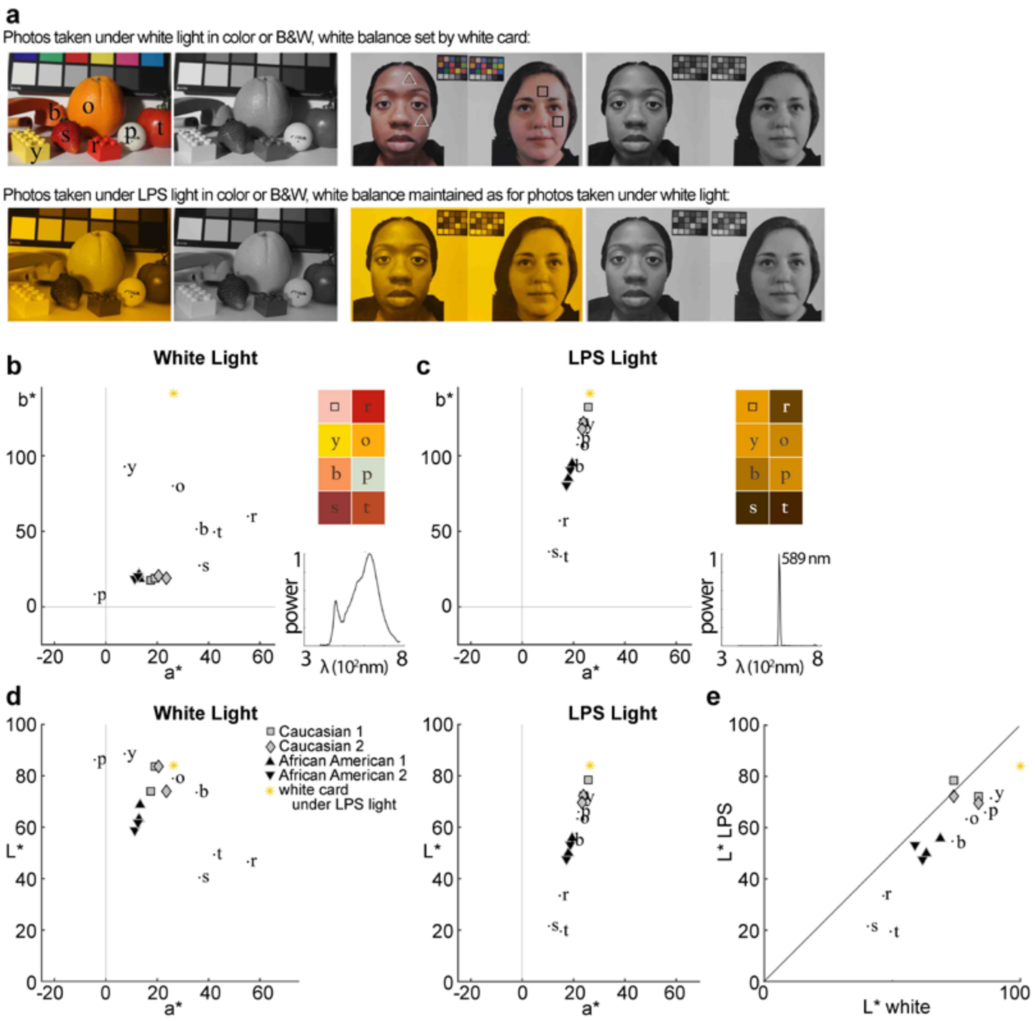

Supplementary Figure 1. Spectral measurements of test stimuli. a, Photographs of the seven objects used, and of two of the actors. The objects are labeled with a letter or a symbol to identify the corresponding spectral measurements given in panels b, c, d, e. The objects were chosen because they occupy the same quadrant as skin in color space. The photographs were taken under white light (top row) and low-pressure sodium light (bottom row), with the white balance set by the white card under white light. The photographs are shown in both color and black and white. **b,** Hue components of spectral measurements of the objects under white light, plotted in a color space designed to be perceptually uniform (CIE L* a* b*). The yellow asterisk shows the Macbeth white card measured under LPS light. Spectral measured XYZ values were transformed to L* a* b* using the XYZ values measured from the Macbeth white card under white light (CIE XYZ = [27.9446 25.0000 10.5134]). Embedded panels show the spectral power distribution of the illuminant (bottom), and the RGB values of the colors of the objects, white balanced using the spectrum of the light measured from the white Macbeth colorchecker card under white light (top). **c,** Hue coordinates of spectral measurements of the objects under low-pressure sodium light white balanced using the spectrum of the light measured from the white Macbeth colorchecker card under white light, as in (b). Other conventions as for panel (b). **d,** Lightness component (L*) of the spectral measurements shown in panels b,c plotted as a function of measured a* component (objects under white light, left; objects under LPS, right). e, Lightness of objects under white light vs LPS light (L* measurements from panel d). Source data are provided as a Source Data file.

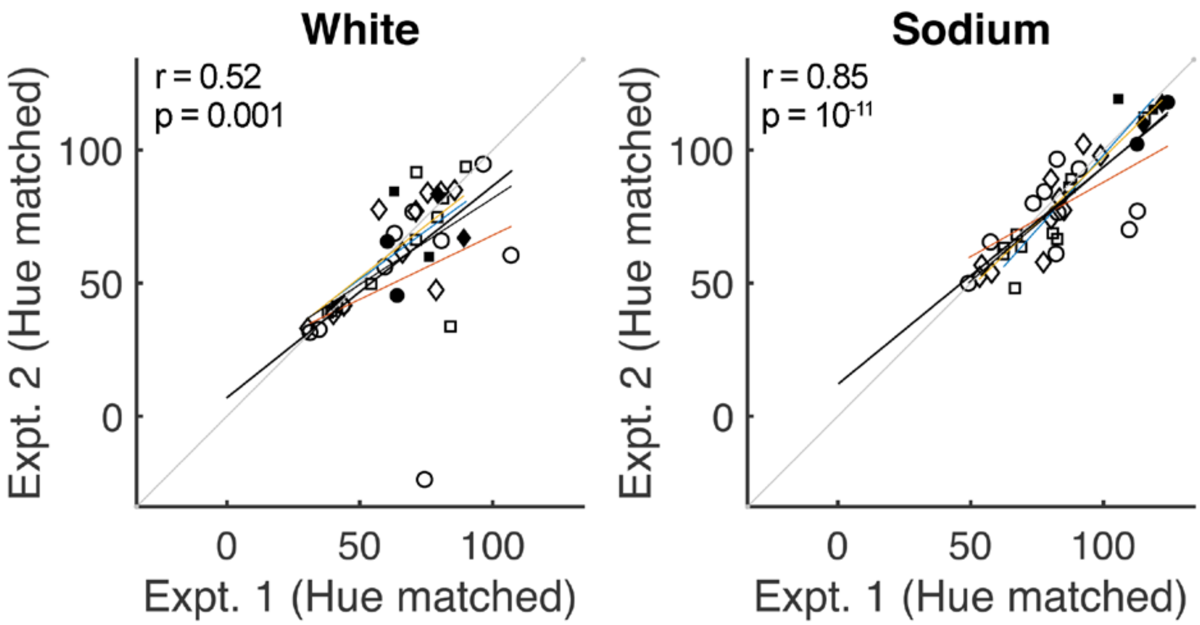

Supplementary Figure 2. Color matches are reliable. Test-retest measurements for three participants, measured three months apart; correlation coefficient and p values are for the population. Regression lines for each participant are shown by the colored lines: Under white light: S1, R^2=0.37 (p=0.036); S2, R^2=0.13 (p=0.24); S3, R^2=0.58 (p=0.004); Under sodium light: S1, R^2=0.88 (p=10^{-6}); S2, R^2=0.46 (p=.002); S3, R^2=0.89 (p=10^{-6}). Source data are provided as a Source Data file.

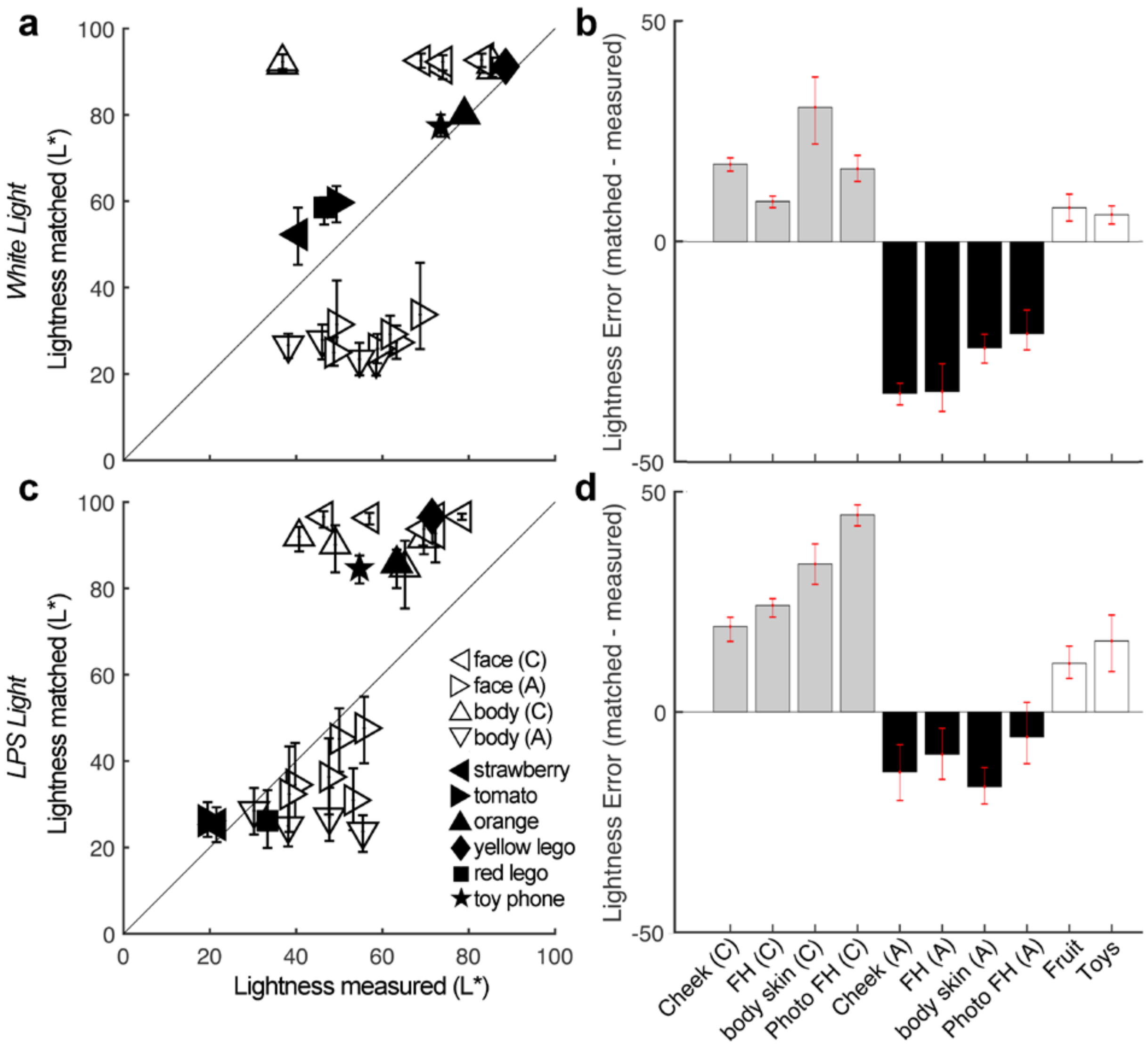

Supplementary Figure 3. Race impacts the lightness of the color matches. a, Matched lightness (CIE L*) versus measured lightness values for objects (filled symbols) and skin (open symbols; key in panel c). **b,** Lightness error (matched minus measured) for different stimuli (axis label in panel d). Error bars are 99 % C.I. Symbols as for Figure 2. **c, d,** as for panels a, and b, but for matches made under low-pressure sodium (LPS) light. Source data are provided as a Source Data file.

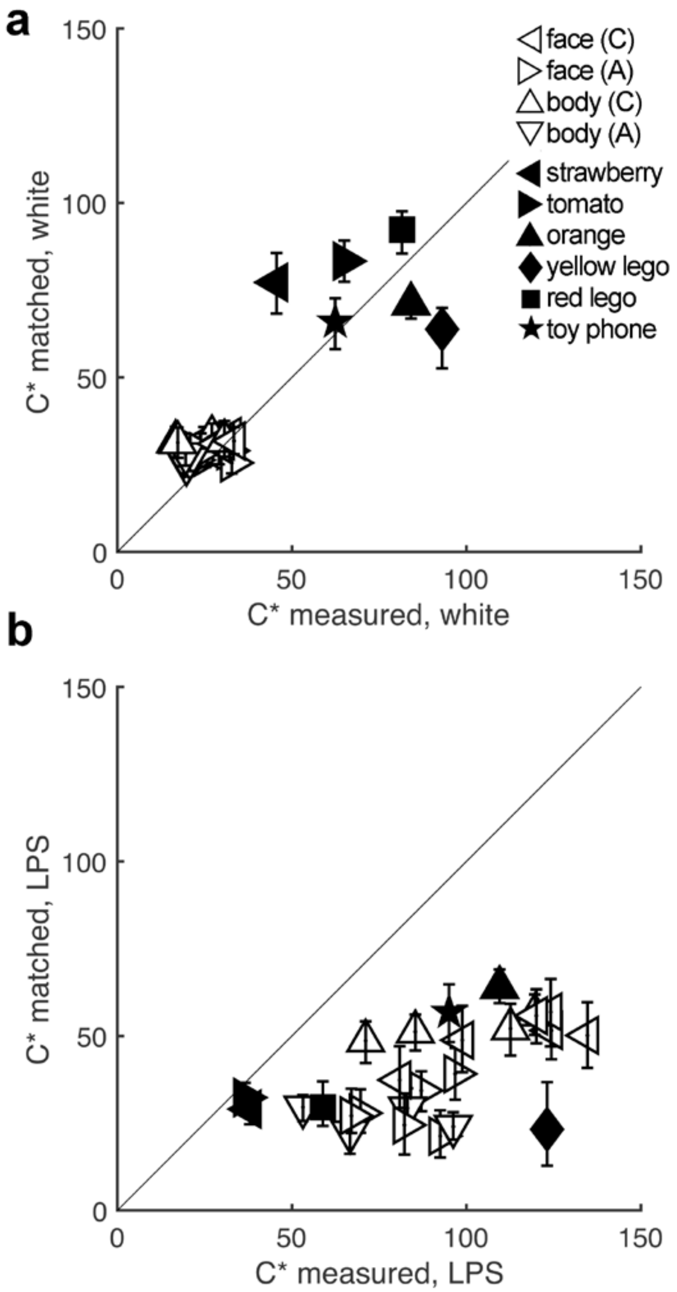

Supplementary Figure 4. Chroma is underestimated under low-pressure sodium light. a, Matched chroma versus measured chroma for objects (filled symbols) and skin (open symbols) under white light. Symbols as for Figure 2. **b,** Matched chroma versus measured chroma for objects (filled symbols) and skin (open symbols) under low-pressure sodium light. Source data are provided as a Source Data file.
