# Supplementary material for: Paradoxical impact of memory on color appearance of faces
Source: Nat Commun. 2019 Jul 8;10:3010. doi: 10.1038/s41467-019-10073-8 (PMC6614425; doi:10.1038/s41467-019-10073-8)
Supplement: Supplementary file 2 — Reporting Summary [file 41467_2019_10073_MOESM2_ESM.pdf]

nature research

Corresponding author(s): Bevil R. Conway
Last updated by author(s): Feb 25, 2019

Reporting Summary

Nature Research wishes to improve the reproducibility of the work that we publish. This form provides structure for consistency and transparency in reporting. For further information on Nature Research policies, see Authors & Referees and the Editorial Policy Checklist.

Statistics

For all statistical analyses, confirm that the following items are present in the figure legend, table legend, main text, or Methods section.

n/a	Confirmed	
☐	☒	The exact sample size (n) for each experimental group/condition, given as a discrete number and unit of measurement
☐	☒	A statement on whether measurements were taken from distinct samples or whether the same sample was measured repeatedly
☐	☒	The statistical test(s) used AND whether they are one- or two-sided<br>*Only common tests should be described solely by name; describe more complex techniques in the Methods section.*
☐	☒	A description of all covariates tested
☐	☒	A description of any assumptions or corrections, such as tests of normality and adjustment for multiple comparisons
☐	☒	A full description of the statistical parameters including central tendency (e.g. means) or other basic estimates (e.g. regression coefficient) AND variation (e.g. standard deviation) or associated estimates of uncertainty (e.g. confidence intervals)
☐	☒	For null hypothesis testing, the test statistic (e.g. F, t, r) with confidence intervals, effect sizes, degrees of freedom and P value noted<br>*Give P values as exact values whenever suitable.*
☒	☐	For Bayesian analysis, information on the choice of priors and Markov chain Monte Carlo settings
☒	☐	For hierarchical and complex designs, identification of the appropriate level for tests and full reporting of outcomes
☒	☐	Estimates of effect sizes (e.g. Cohen's d, Pearson's r), indicating how they were calculated

Our web collection on statistics for biologists contains articles on many of the points above.

Software and code

Policy information about availability of computer code

Data collection	Data was collected with MATLAB 2016 and Psychtoolbox-3.
Data analysis	Data was analyzed with MATLAB R2017b. All analysis code and raw data are available at: https://neicommons.nei.nih.gov/#/facecolor

For manuscripts utilizing custom algorithms or software that are central to the research but not yet described in published literature, software must be made available to editors/reviewers. We strongly encourage code deposition in a community repository (e.g. GitHub). See the Nature Research guidelines for submitting code & software for further information.

Data

Policy information about availability of data

All manuscripts must include a data availability statement. This statement should provide the following information, where applicable:

- Accession codes, unique identifiers, or web links for publicly available datasets
- A list of figures that have associated raw data
- A description of any restrictions on data availability

Data and analysis codes are available at https://neicommons.nei.nih.gov/#/facecolor

Field-specific reporting

Please select the one below that is the best fit for your research. If you are not sure, read the appropriate sections before making your selection.

☐ Life sciences ☒ Behavioural & social sciences ☐ Ecological, evolutionary & environmental sciences

For a reference copy of the document with all sections, see nature.com/documents/nr-reporting-summary-flat.pdf

Behavioural & social sciences study design

All studies must disclose on these points even when the disclosure is negative.

Study description	We conducted a quantitative experimental study (visual psychophysics in a normal population). Data collected were both quantitative (participants made color appearance matches from a complete digital color gamut) and qualitative (verbal reflections on the experience of the experimental condition).
Research sample	Participant information (N=20): average age 27.5 (range 19-33);10 female; 10 male; 12 Caucasian; 4 Asian American; 3 African American; 1 South-East Asian American; all participants were residents of the Boston area; all participants had normal color vision.
Sampling strategy	Sampling was random. Prior to data collection, we sought to recruit a gender-balanced participant population of N=20, between the ages of 18-35, and with normal color vision. A sample size of 20 was chosen based on an initial pilot experiment involving separate participants and actors that provided the basis for the experimental approach reported here. Participants were recruited on line via a volunteer mailing list operated by the Department of Brain and Cognitive Sciences at MIT, and were screened for normal color vision (with Ishihara plates).
Data collection	Twenty people with normal color vision (tested with Ishihara plates) participated in the experiment. Each participant matched the color appearance of seven real-world objects including four artificially colored toys (orange phone, red Lego, yellow Lego, white ping-pong ball), three ripe fruits (strawberry, tomato, orange), and various skin regions of four actors (all female; 2 Caucasian, 2 African American; no makeup). Color appearance matches were made using a calibrated computer monitor encased in a light-tight box, inside the testing room. Participants could see the monitor by lifting a small black cloth to reveal a 2cm2 viewing window. The monitor showed a color-space disc and a lightness bar that the participant could use to adjust a central patch to make their selection with a computer mouse. The monitor was otherwise black, and the color of the test patch was random at the onset of testing. Participants were instructed to match the color of the patch as accurately as possible to the color appearance of the test stimuli, and not to match the color of the stimuli as recalled from memory. The participants did the matching twice: first while the testing room was illuminated with LPS light, and then again after the room light was switched to normal white light (participants adapted to the illumination for ~7 minutes). Participants were tested first under LPS light to prevent short-term recall of color matches made under white light. Test stimuli were presented in a unique order for each participant (SI Table 1) at ~1m viewing distance. The precise region to be matched was indicated with a lightly drawn circle (for the objects) or by pointing to regions on the actors (forehead, lips, neck, and back of the hand). Participants also color-matched the forehead of each actor in a photograph and the actors' foreheads while masking the rest of the face with black paper (~1 cm diameter aperture). All participants matched the same region of each stimulus, and the lighting on the stimuli was consistent across participants: stimuli were placed at a set location on a table, at a fixed distance from the light sources; actors were seated with their faces at a fixed orientation and gaze angle with respect to the participant. The actors entered the testing room one at a time. The participant's adaptation state was preserved during transitions by having the participant close their eyes and by keeping the anteroom dark. An experimenter, who was aware of the study hypotheses, was present in the testing room during each session in order to physically swap out each stimulus as the experiment progressed, and was therefore not blind to the experimental conditions. After participants completed the matches under LPS light and before they proceeded to the tasks under white light, the experimenter asked participants to "tell us if you noticed anything about your color experience". The experimenter transcribed participant comments in a lab notebook. Spectral measurements of the stimuli and matching monitor were obtained with a spectroradiometer (PR655, Chatsworth, CA).
Timing	Data were collected June 1st-July 31st 2016.
Data exclusions	No participant's data was excluded from the analyses.
Non-participation	No participants declined participation in the study
Randomization	Participants were not allocated into experimental groups. Test stimuli were presented in a unique order for each participant (SI Table 1), assigned at random.

Reporting for specific materials, systems and methods

We require information from authors about some types of materials, experimental systems and methods used in many studies. Here, indicate whether each material, system or method listed is relevant to your study. If you are not sure if a list item applies to your research, read the appropriate section before selecting a response.

Materials & experimental systems

n/a	Involved in the study	
☒	☐	Antibodies
☒	☐	Eukaryotic cell lines
☒	☐	Palaeontology
☒	☐	Animals and other organisms
☐	☒	Human research participants
☒	☐	Clinical data

Methods

n/a	Involved in the study	
☒	☐	ChIP-seq
☒	☐	Flow cytometry
☒	☐	MRI-based neuroimaging

Human research participants

Policy information about studies involving human research participants

Population characteristics	See above
Recruitment	Participants were recruited on line via a volunteer mailing list operated by the Department of Brain and Cognitive Sciences at MIT. List subscribers are not necessarily MIT-affiliates, but it is likely that a majority are.
Ethics oversight	All experimental procedures were approved by the Wellesley College Institutional Review Boards, the Massachusetts Institute of Technology Committee on the Use of Humans as Experimental Subjects, and the National Institutes of Health Intramural Institute Clinical Research Review Committee. All participants and actors provided written informed consent and were compensated financially for their involvement. The two actors whose photographs are shown in Supplementary Figure 1 provided written consent to publish their photographs

Note that full information on the approval of the study protocol must also be provided in the manuscript.
