# Supplementary material for: Paradoxical impact of memory on color appearance of faces
Source: Nat Commun. 2019 Jul 8;10:3010. doi: 10.1038/s41467-019-10073-8 (PMC6614425; doi:10.1038/s41467-019-10073-8)
Supplement: Supplementary file 3 — Description of Additional Supplementary Information [file 41467_2019_10073_MOESM3_ESM.pdf]

Description of Additional Supplementary Files

File Name: Supplementary Data 1
Description: This file contains the CIE L* a* b* values of participants’ color matches to real three-dimensional stimuli presented under (1) low-pressure sodium Illumination and (2) broadband white illumination. The table also contains spectral measurements of the stimuli. The file is comprised of two data sheets: the first tab contains the data from the main experiment (N=20 subjects; 35 stimuli); the second tab contains data from a follow-up experiment (N=3 subjects; 20 stimuli, 14 repeat stimuli, 6 novel; data presented in Figure 6 & SI Figure 2).

File Name: Supplementary Data 2
Description: This file contains the unique stimulus presentation orders for each subject.
